# Mesenchymal stem cells in multiple myeloma: a therapeutical tool or target?

**DOI:** 10.1038/s41375-018-0061-9

**Published:** 2018-02-22

**Authors:** Song Xu, Kim De Veirman, Ann De Becker, Karin Vanderkerken, Ivan Van Riet

**Affiliations:** 1 Department of Lung Cancer Surgery, Lung Cancer Institute, Tianjin Medical University General Hospital, Tianjin, China; 2 Department Hematology- Stem Cell Laboratory, Universitair Ziekenhuis Brussel (UZ Brussel), Brussels, Belgium; 3 Research Group Hematology and Immunology-Vrije Universiteit Brussel (VUB), Myeloma Center Brussels, Brussels, Belgium

## Abstract

Multiple myeloma (MM) is a malignant plasma cell (PC) disorder, characterized by a complex interactive network of tumour cells and the bone marrow (BM) stromal microenvironment, contributing to MM cell survival, proliferation and chemoresistance. Mesenchymal stem cells (MSCs) represent the predominant stem cell population of the bone marrow stroma, capable of differentiating into multiple cell lineages, including fibroblasts, adipocytes, chondrocytes and osteoblasts. MSCs can migrate towards primary tumours and metastatic sites, implying that these cells might modulate tumour growth and metastasis. However, this issue remains controversial and is not well understood. Interestingly, several recent studies have shown functional abnormalities of MM patient-derived MSCs indicating that MSCs are not just by-standers in the BM microenvironment but rather active players in the pathophysiology of this disease. It appears that the complex interaction of MSCs and MM cells is critical for MM development and disease outcome. This review will focus on the current understanding of the biological role of MSCs in MM as well as the potential utility of MSC-based therapies in this malignancy.

## Introduction

Multiple myeloma (MM) is a haematological malignancy characterized by a clonal proliferation of plasma cells in the bone marrow (BM) and the presence of monoclonal immunoglobulin in the blood and/or urine. A major characteristic of this disease is the predominant localization of MM cells in the BM. The crosstalk between BM stromal cells and MM cells supports the proliferation, survival, migration and drug resistance of MM cells, as well as osteoclastogenesis and angiogenesis. Mesenchymal stem cells (MSCs) are self-renewing and multipotent progenitors that can differentiate into a variety of cell types, such as adipocytes, endothelial cells, osteoblasts and fibroblasts, which constitute the main cellular compartment of BM stroma. Many studies have demonstrated that MSCs play an important role in the growth of different tumour types. As the precursors of BM stromal cells, MSCs are thought to be involved in the pathophysiology and progression of MM as well. Moreover, MM patient-derived MSCs (MM-hMSCs) seem to be genetically and functionally different compared to MSCs derived from normal donors (ND-hMSCs). Currently, there is increasing interest in using MSCs for therapeutic applications in cancer patients. In particular, clinical trials have been initiated to evaluate the clinical potential of donor-derived MSCs to control steroid-resistant graft versus host disease after allogeneic haematopoietic stem cell (HSC) transplantation and to support HSC engraftment after both autologous and allogeneic transplantation in patients with various haematological malignancies, including MM. Here, we review the current understanding of the possible role of MSCs, both in the biology and the treatment of MM.

## Abnormalities of MSCs in MM

MSCs are an essential cell type in the formation and function of the BM microenvironment, and several previous studies have evaluated the difference between MM-hMSCs and ND-hMSCs. Regardless of the disease stage, the surface immunophenotype of MM-MSCs was similar to that from ND-MSCs [1–4]. Garderet el al. [3] reported that MM-MSCs exhibited a much lower proliferative capacity than ND-MSCs, associated with a reduced expression of the receptors for platelet-derived growth factor-α and -β, insulin-like growth factor-1, epidermal growth factor and basic fibroblast growth factor (bFGF). The growth impairment was more pronounced in MM patients with advanced disease and bone lesions [5]. In contrast, Corre et al. [2] showed that the expansion of BM MSCs was not different among normal donors, monoclonal gammopathy of undetermined significance (MGUS) patients and MM patients.

Compared with their normal counterparts, MM-MSCs differ in their spontaneous and myeloma cell-induced production of cytokines. MM-MSCs can express abnormally high mRNA and protein levels of interleukin (IL)-6, which is the most potent growth factor involved in MM progression [1–4]. Dickkopf-1 (DKK1) production was also found to be enhanced in MM-MSCs [2, 3]. In addition, MM-MSCs can constitutively express high amounts of IL-1β, IL-3, granulocyte-colony stimulating factor (CSF), granulocyte monocyte (GM)-CSF, stem cell factor and tumour necrosis factor (TNF)-α [1–4]. Zdzisinska et al. [5] observed that MM-MSCs had a higher capacity to produce IL-6, IL-10, TNF-α, osteopontin and especially hepatocyte growth factor (HGF) and B cell-activating factor than ND-MSCs in the presence of RPMI 8226 MM cells (under cell-to-cell contact as well as non-contact conditions). The authors of this study also found that MM-MSCs significantly enhanced the production of sIL-6R by the RPMI 8226 MM cells [5]. In addition, Corre et al. [2] observed that MSCs from MM patients overexpressed growth differentiation factor 15 (GDF15) [2]. Recent studies suggested that GDF15 contributes to myeloma cell growth and chemoresistance and, even more importantly, that high levels of GDF15 are correlated with a poor prognosis in MM patients [6]. André et al. [7] demonstrated that MM BM-derived MSCs exhibited an increased expression of senescence-associated β-galactosidase, increased cell size, reduced proliferative capacity and characteristic expression of senescence-associated secretory profile members compared to the normal counterparts. This senescent state most likely participates in disease progression and relapse by altering the tumour microenvironment [7].

Why do MSCs from MM patients express abnormal cytokines favouring MM progression? Using microarray analysis, Corre et al. [2] have observed a distinctive gene expression profile between MM-MSCs and ND-MSCs, with differential expression of genes coding for growth and angiogenic factors, as well as for factors related to bone differentiation. All of these differences were detected after isolation and expansion of MSCs in culture. Garayo et al. [8] observed that cultured MM-MSCs show a distinctive array-comparative genomic hybridization profile compared to that observed in their normal counterparts. However, to which extent these molecular aberrations in MM-MSCs may have an impact on their function and, thus, on the progression/relapse of MM disease still remains to be determined [8]. McNee et al. [9] recently identified that peptidyl arginine deiminase 2 (PADI2) was one of the most highly upregulated transcripts, in MSCs from both MGUS and MM patients, that could induce upregulation of IL-6 through its enzymatic deimination of histone H3 arginine 26. Li et al. [10] also found that MM-MSCs had a significantly longer telomere length, which was positively associated with the expression of IL-6 and chemokine (C-C motif) ligand 3 (CCL3).

Some evidence also suggested that the abnormalities of MM-MSCs could be acquired through exposure to myeloma cells. MM cells could reduce the expression of miR-223 and miR-485-5p in vitro, which altered the senescence phenotype of MM-MSCs with participation of the delta-like homologue 1- iodothyronine deiodinase 3 (DLK1-DIO3) genomic region [11]. Co-cultivation of MM cells and MM-MSCs induced a reduced miR-223 expression and activation of Notch signalling in MM-MSCs, leading to increased vascular endothelial growth factor and IL-6 expression and impaired osteogenic differentiation potential [12]. Our group uncovered that MM-hMSCs have a different microRNA (miRNA) expression profile compared to their normal counterparts. Using bioinformatics tools, we found that some differentially expressed miRNAs were possibly relevant to some functional abnormalities of MM-hMSCs, for example, the impaired osteogenic differentiation [13]. We also observed that MM cell-derived soluble factors could induce an upregulation of miR-135b expression in ND-hMSCs in an indirect coculture system. Targeting these miRNAs might help in correcting the MM tumour microenvironment.

In addition, Todoerti et al. [14] further determined that BM-MSCs compared to osteoblasts had distinct transcriptional profiles in multiple myeloma bone disease. Wang et al. [15] demonstrated that the mRNA and protein levels of angiogenic factors were elevated in MSCs derived from multiple myeloma compared with normal donors. Li et al. [16] also reported that MSCs from MM patients showed impaired immunoinhibitory capability on T cells. André et al. [17] demonstrated that altered immunomodulation capacities of MM BM-MSCs were linked to variations in their immunogenicity and secretion profile including IL-6, vascular cell adhesion molecule 1 (VCAM-1) and CD40. These alterations lead not only to a reduced inhibition of T-cell proliferation but also to a shift in the T helper 17 cell/ regulatory T-cell balance [17]. Furthermore, Pevsner-Fischer et al. [18] recently observed that MM-hMSCs exhibited a different expression of extracellular-regulated kinase-1/2 phosphorylation in response to Toll-like receptor ligands and epidermal growth factor compared with ND-hMSCs. Evidence also showed that MM-MSCs, in contrast to ND-MSCs, could produce a higher amount of immune-modulatory factors that are involved in granulocytic-myeloid-derived suppressor cell (G-MDSC) induction. MM-MSCs stimulate G-MDSCs to upregulate immune-suppressive, angiogenesis and inflammatory factors as well as to digest bone matrix [19]. Previous studies also indicated that MM-MSCs demonstrated other abnormalities, including distinct histone deacetylase (HDAC) expression patterns [20], upregulated thymic stromal lymphopoietin expression and induction of T helper 2 cell responses [21], a constitutively high level of phosphorylated Myosin II conferring enhanced collagen matrix remodelling and promoting the MM and MSC interaction [22], and a stiffer phenotype (biomechanical changes) induced by MM cells [23].

**Fig. 1:**
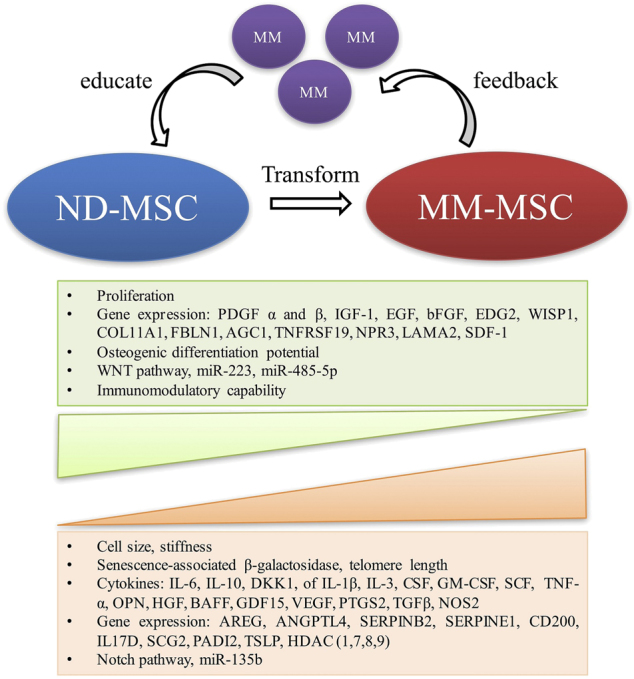
Biological alterations of MSCs in the MM tumour microenvironment. It can be assumed that normal MSCs are educated by MM cells and transform into MM-MSCs, which in turn influence MM cell growth. It cannot be excluded that some abnormalities are intrinsic (and not MM cell-induced). MSC mesenchymal stem cell, MM multiple myeloma, PDGF platelet-derived growth factor, IGF1 insulin-like growth factor 1, EGF epidermal growth factor, bFGF basic fibroblast growth factor, EDG2 endothelial differentiation, lysophosphatidic acid G-protein-coupled receptor 2, WISP1 WNT1-induced secreted protein-1, COL11A1 collagen type XI α1 chain, SDF1 stromal derived factor-1, FBLN1 fibulin 1, AGC1 amino acid transporter AGC1, TNFRSF19 TNF receptor superfamily member 19, NPR3 natriuretic peptide receptor 3, LAMA2 laminin subunit α2, IL interleukin, DKK1 dickkopf-1, CSF colony stimulating factor, SCF stem cell factor, TNF-α tumour necrosis factor-α, OPN osteopontin, HGF hepatocyte growth factor, VEGF vascular endothelial growth factor, BAFF B cell-activating factor, GDF15 growth differentiation factor 15, PTGS2 prostaglandin-endoperoxide synthase 2, TGFβ transforming growth factor-β, NOS2 nitric oxide synthase 2, AREG amphiregulin, ANGPTL4 angiopoietin like 4, SERPINB2 serpin family B member 2, SERPINE1 serpin family E member 1, SCG2 secretogranin II, PADI2 peptidyl arginine deiminase 2, TSLP thymic stromal lymphopoietin, HDAC histone deacetylase

All of the data summarized above demonstrate that MM-MSCs are genetically and functionally different from MSCs in healthy subjects (Fig. 1). All of these MM-MSC abnormalities, either intrinsic or inducible, lead to the formation of a more favourable BM microenvironment for MM tumour development and progression.

## Effects of MSCs on tumour growth in MM

**Fig. 2:**
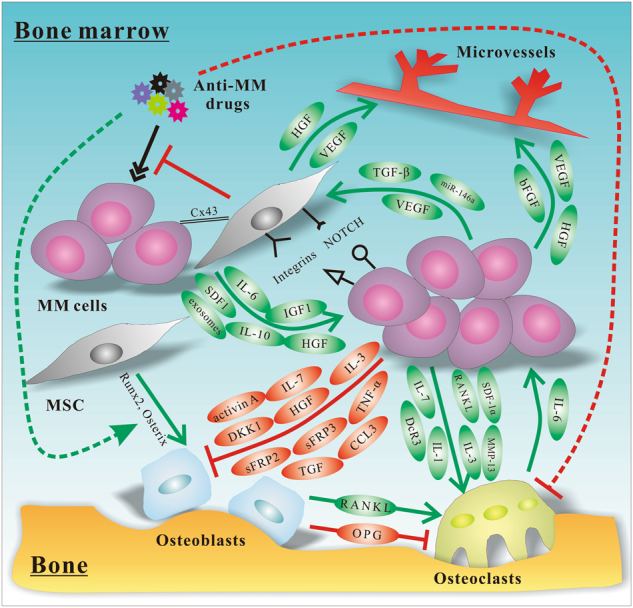
Schematic diagram of MSC interactions in MM tumour microenvironment. Direct and indirect interactions with MM cells induce MSCs to acquire abnormal phenotypes, which in turn lead to the formation of BM microenvironment influencing MM tumour development and progression of osteolytic bone lesions. MSC mesenchymal stem cell, MM multiple myeloma, HGF hepatocyte growth factor, VEGF vascular endothelial growth factor, TGF-β transforming growth factor-β, bFGF basic fibroblast growth factor, IL interleukin, DKK1 Dickkopf-1, Cx43 Connexin-43, SDF1 stromal-derived factor-1, IGF1 insulin-like growth factor 1, TNF-α tumour necrosis factor-α, sFRP secreted frizzled-related protein, CCL3 chemokine (C-C motif) ligand 3, RANKL nuclear factor-κB ligand, DcR3 soluble decoy receptor 3, MMP-13 matrix metalloproteinases 13, OPG osteoprotegerin, Runx2 runt-related transcription factor 2

Stephen Paget proposed the ‘‘seed and soil’’ hypothesis in 1889, indicating that the establishment of tumour metastatic sites is influenced by cross-interaction between selected cancer cells (‘‘seed’’) and specific organ microenvironments (‘‘soil’’). With regard to MM, tumour cells grow predominantly in BM and the cellular and non-cellular components of the MM BM microenvironment play an essential role in supporting MM cell proliferation, survival, migration and chemoresistance [24]. Evidence has been provided that MSCs give rise to most BM stromal cells that interact with MM cells, and are involved in the pathophysiology of MM (Fig. 2).

Some studies have shown that interactions between MSCs and MM cells support the proliferation of myeloma. MSCs strongly support MM cell growth by the production of high levels of IL-6, a major MM cell growth factor [25]. MM cells secrete DKK1, which prevents MSCs from differentiating into osteoblasts, and the undifferentiated MSCs can produce IL-6, which in turn stimulates the proliferation of DKK1-secreting MM cells [26]. It has been assumed that direct contact between the two types of cells, partially mediated through the very late antigen-4 (VLA-4) and RGD-peptide mechanisms, was necessary for this induction [25]. In another study, it was found that a significant increase in IL-6 concentration occurred when the myeloma cells were cocultured with BM MSCs of MM patients in a non-contact Transwell system. It was suggested that bFGF, secreted by myeloma cells, bound to bFGF receptors on BM MSCs and thus stimulated IL-6 production [27]. Recent evidence showed that survivin was involved in an anti-apoptotic effect of MSCs on myeloma cells [28]. Direct contact with MSCs was also found to influence MM cell growth and the MM cell phenotype [29]. Furthermore, the crosstalk between BM MSC and MM cells was reported to support osteoclastogenesis and angiogenesis in MM [30–32]. In our study, we found that MSCs had tropism towards MM cells, and CCL25 was identified as a major MM cell-produced chemoattractant. MSCs favoured the proliferation of MM cells and protected them against spontaneous and Bortezomib-induced apoptosis in vitro and in vivo. Infusion of in vitro expanded murine MSCs in 5T33 MM mice resulted in a significantly shorter survival [33]. Kim et al. [34] also reported that MM cells, U266 and NCI-H929, exhibited an increased proliferation and a decreased apoptosis rate in the presence of MSCs, which was consistent with our in vitro findings. Noll et al. [35] showed that MSCs derived from bone marrow in myeloma patients, expressed higher levels of the plasma cell-activating factor IL-6 and the osteoclast-activating factor receptor activator of nuclear factor-κB ligand (RANKL) [35]. Dotterweich et al. [36] demonstrated that MSC contact promoted angiogenic factor CCN family member 1 (CCN1) splicing and transcription in MM cells, which favoured tumour viability and myeloma bone disease. Roccaro et al. [37] recently described a mechanism whereby MM-derived BM-MSCs contributed to MM disease progression in vitro and in vivo via released exosomes. Our group also reported that MM cells can transfer miR146a through exosomes and promote the increase in cytokine secretion in MSCs, which in turn favoured MM cell growth and migration [38]. Moreover, the Bruton tyrosine kinase (BTK) signal pathway and Connexin-43 were also involved in the MSC and MM cell interaction, which increased myeloma stemness and tumour cell proliferation [39, 40].

However, some studies described opposite findings. Using a SCID-rab MM mouse model, Li et al. [41, 42] demonstrated that both MSCs and placenta-derived adherent cells, which are mesenchymal-like stem cells isolated from postpartum human placenta, effectively suppress bone destruction and tumour growth in vivo, although these stem cells significantly support MM cell growth in vitro [41, 42]. Ciavarella et al. [43] also found that, in contrast to BM-derived MSCs, adipose tissue-derived MSCs and umbilical cord-derived MSCs (UC-MSCs) significantly inhibited MM cell clonogenicity and growth in vitro and in vivo. They proposed that UC-MSCs have a distinct molecular profile compared with other MSCs and exhibit a different effect on MM cells [43]. Atsuta et al. [44] demonstrated that Fas/Fas-L-induced MM apoptosis played a crucial role in the MSC-based inhibition of MM growth [44]. In addition, there are also some reports showing that MSCs have no significant effect on MM growth [45–47].

Potential explanations were reported regarding the different effects of MSCs on MM tumour growth. Kanehira et al. [48] explored the role of lysophosphatidic acid (LPA) signalling in cellular events where MSCs were converted into either MM-supportive or MM-suppressive stroma. They found that myeloma cells stimulate MSCs to produce autotaxin, an essential enzyme for the biosynthesis of LPA. LPA receptor 1 (LPA1) and 3 (LPA3) transduce opposite signals to MSCs to determine the fate of MSCs. LPA1-silenced MSCs showed a delayed progression of MM and tumour-related angiogenesis in vivo, while LPA3-silenced MSCs significantly promoted the progression of MM and tumour-related angiogenesis in vivo. Therefore, during the different stages and conditions, MSCs might exhibit different effects on MM growth [48]. In addition, it can be assumed that several factors such as the nature of the in vivo MM model (severe combined immunodeficiency (SCID) or non-SCID mice), the MM cell lines used as well as the number and source of the injected MSCs and the timing of injection might all account for the discrepancy between these studies. The details of the different observations about MSC effect on MM growth are listed in Table 1.

**Table 1:** Studies about MSC effects on MM growth

**Authors**	**Source of isolation**	**MM cell**	**MM model**	**MSC/MM cell ratio and timing of infusion**		**Findings**	
				In vitro	In vivo	In vitro	In vivo
Li et al. [41]	Human placenta-derived adherent cell (PDAC) and BM-derived MSCs	Primary MM cells and MM cell lines (BN, JB, ARP1, U266, DNC, HLE, H929)	SCID-rab model (i.b. injection with primary MM cells or H929 myeloma cell line) and SCID mice (injected s.c. with H929 myeloma cell line)	1:1, for 7 days	SCID-rab model: 1 × 10^6^ MM cells per bone, 3 weeks later, 1 × 10^6^ PDAC or MSCs per bone	MSCs and PDACs supported growth of myeloma cells.	PDACs or MSCs inhibit ed MM growth in bone but not in subcutaneous microenvironment
					SCID mice: 5 × 10^6^ MM cells s.c., 4 weeks later, 1 × 10^6^ PDAC or MSCs s.c.		
Li et al. [42]	Human BM-derived MSCs	Hg myeloma cells	SCID-hu and SCID-rab MM model	N/A	0.5 × 10^6^ Hg myeloma cells per bone, when high levels were >1.5 µg/ml, 1 × 10^6^ MSCs were injected i.b. or i.v., single or four weekly	N/A	Injection i.b. of MSCs inhibited MM growth in bone. But myeloma growth was not affected by either single or sequential i.v. injections of MSCs.
Ciavarella et al. [43]	Human umbilical cord-derived MSCs (UC-MSCs)	RPMI8226	NOD.CB17-Prkdc^scid^/J mice	2:1, for 72 h	1 × 10^6^ RPMI-8226 cells plus UC-MSCs at a 1:2 ratio subcutaneously; Or peritumoral injections of 2 × 10^6^ UC-MSCs	UC-MSCs significantly suppressed MM cells growth and clonogenicity.	Co-injections of RPMI-8226 with UC-MSCs or peritumoral inoculations of UC-MSCs resulted in evident inhibition of tumour growth
Atsuta et al. [44]	Murine BM-derived MSCs	5TGM1 MM cell line	5TGM1 MM model	1:1, 1:5, 1:10, 24 h	6 × 10^6^ 5TGM1 cells i.v. inoculation, 1 week later, MSCs i.v. injection (1 × 106 MSCs/10 g body weight)	MSCs inhibited MM cell growth	The administration of MSCs prolonged survival compared with the MM group, and resulted in a decrease of lung metastasis.
Xu et al. [33]	Human- and murine BM-derived MSCs	Murine 5T33MMvv and 5T33MMvt cells, human MM cell lines (RPMI8226, MM5.1)	5T33 MM model	1:5–1:100, 24 or 48 h	Injection i.b.: 1:10 co-injection for 1 or 2 weeks	MSCs protected MM cells against apoptosis and favoured stroma-dependent MM cells growth	MSCs favored MM cell growth in vivo and decrease MM mice survival.
					Injection i.v.: after inoculation of 1 × 10^5^ 5T33MMvv cells on day 0, 2 × 10^5^ MSCs were injected i.v. on days 6, 10 and 14.		
Kim et al. [34]	Human BM MSCs	Human MM cell lines (U266 and NCI-H929)	N/A	72 h, ratio is not mentioned.	N/A	MSCs stimulated the proliferation and decreased apoptosis of MM cell.	N/A
Roccaro et al. [37]	MM and normal BM-MSCs, murine BM-MSCs	MM.1S, RPMI8226, and U266	miR-15a/16-1^–/–^ mice	MM cells were exposed in MSC-derived exosomes up to 48 h	3 × 10^6^ MM cells and 1 μg BM-MSC–derived exosomes co-injected, and 1 μg exosomes were injected in situ every 4 days up to 14 days	MM BM MSC-derived exosomes increased MM cell proliferation.	MM BM MSC-derived exosomes increased MM tumour growth.
Ciavarella et al. [45]	Adipose-derived MSCs (AD-MSCs)	Human MM cell lines (RPMI8226, U266, CAR1, LIG1, MCC2)	N/A	1:1 or 2:1, for 24 h	N/A	Unmanipulated MSCs had no significant effect on MM proliferation	N/A
Sartoris et al. [46]	Murine MSCs	Sp6 cells	Sp6 xenogeneic model (Engraftment of Sp6 cells into Balb/c mice s.c.)	1:1, for 5 days	1:1 s.c. 1 or 3 doses	Unmanipulated MSCs had no significant effect on tumour cell survival.	Unmanipulated MSCs had no significant effect on tumour growth and tumour bearing mice survival.
Gunn et al. [26]	Human BM MSCs	Human MM cell lines (XG1 and ANBL1)	N/A	MM cells were exposed in MSCs conditioned medium up to 4 days	N/A	The proliferation of MM cells increased significantly when exposed in MSC conditioned medium.	N/A
Rabin et al. [47]	Human BM MSCs	KMS-12-BM	KMS-12-BM xenogeneic MM model (Engraftment of KMS-12-BM in 2m NOD/SCID mice)	N/A	1 × 10^6^ MSC^OPG^, MSC^GPO^ or unmodified MSCs were injected by tail vein into β_2_m NOD/SCID mice 2, 3 and 4 weeks after administration of 1 × 10^7^ MM cell KMS-12-BM	N/A	There was no significant difference in tumour take between those groups that did and did not receive MSC.
Kanehira et al. [48]	Human BM MSCs	OPM-2, IM-9 and RPMI-8226	BALB/c-nu/nu mice	N/A	1 × 10^6^ IM-9 cells were cotransplanted with 4 × 10^5^ MSCs silenced with siRNAs against LPA1, LPA3 into the left flank	N/A	siLPA3-MSCs promote MM progression and angiogenesis, while siLPA3-MSCs delayed MM progression and inhibited tumour-related angiogenesis.

*i.v.* intravenous, *i.b.* intrabone, *s.c.* subcutaneous

## Role of MSCs in bone disease

MM is the disease with the highest incidence of bone involvement among all the malignant diseases. Abnormalities in conventional radiography can be found in approximately 80% of patients with newly diagnosed MM. Bone disease in MM is characterized by lytic bone lesions, which can cause severe bone pain, pathologic fractures and hypercalcaemia. The basic mechanism of increased bone resorption in MM is an uncoupling of normal bone remodelling with increased osteoclast activity and decreased osteoblast function [26].

In MM patients, increased numbers of osteoclasts are present at sites of bone destruction. These osteoclasts are stimulated by local osteoclast-activating factors that are produced by myeloma cells and/or cells of the bone microenvironment. The RANK/RANKL/osteoprotegerin (OPG) system has been widely studied in bone remodelling. It has been demonstrated that in MM an increased RANKL/OPG ratio results in enhanced osteoclast formation and activation, which is a major mechanism in MM-related bone disease [49]. Several other factors have also been identified as main osteoclast inducers in MM: the chemokines CCL3 and CCL4, stromal-derived factor-1α, soluble decoy receptor 3 (DcR3), matrix metalloproteinases (MMP)-13, IL-1, IL-3, IL-6 and IL-17 [50–65]. Moreover, adhesive interactions between MM and stromal cells also play a significant role in promoting osteoclastogenesis and augmenting the bone destructive process [66]. In addition, myeloma cells can adhere directly to osteoclasts, resulting in increased myeloma cell proliferation and osteoclastic differentiation [67, 68]. The mechanisms that have been described to be involved in MM bone disease are listed in Table 2.

**Table 2:** Mechanisms involved in MM bone disease and therapeutic potentials

**Osteoblastogenesis inhibition**		**Osteoclastogenesis activation**	
Factors	Therapeutic strategy	Factors	Therapeutic strategy
VLA-4 /VCAM-1	Anti-α4 integrin antibody [70]	RANKL	Anti-RANKL antibody; OPG [50, 51]
NCAM	Anti-NCAM antibody [72]	MIP-1α/ MIP-1β	CCR1/CCR5 inhibitor; anti MIP-1α/ MIP-1β antibody [52, 91]
DKK1	Anti-DKK1 antibody; GSK-3β inhibitor [73, 74]	SDF-1	CXCR4 inhibitor [54]
MIP-1α	CCR1 inhibitor [91]	IL-3	Anti-IL-3 antibody [55]
IL-3	Anti-IL-3 antibody [84]	IL-6	Anti-IL-6 antibody [56]
IL7	Anti-IL-7 antibody [70, 81]	TNF-α	Anti–TNF-α antibody; Antagonist of NF-κB activation [59, 60]
sFRPs	Anti-sFRPs antibody; GSK-3β inhibitor [76, 77]	BDNF	TrkB inhibitor [61]
TGF-β	TGF-β receptor inhibitor [82]	IL7	Anti-IL-7 antibody [70, 81]
Activin A	Activin A receptor antagonist [86]	IL-17A	Anti-IL-17 antibody [62, 63]
Gfi1	Anti–TNF-α antibody or Gfi1 siRNA [89]	IL-1	Anti-IL-1 antibody [65]
Sclerostin	Sclerostin antagonist [78, 79]	DcR3	Anti-DcR3 antibody [58]
HGF	c-Met inhibitor [83]	MMP-13	MMP-13 knockdown [64]
Notch	gamma Secretase inhibitor [69]		
miR-135b	miR-135b antagomir [13]		
Ror2	Overexpression of Wnt5 or Ror2 by lentiviral vectors [80]		
lncRNA MEG3	Overexpression of MEG3 by lentiviral vectors [92]		

*VLA-4* very late antigen-4/ integrin α4β1, *VACM-1* vascular cell adhesion protein 1, *NCAM-1* neural cell adhesion molecule-1,* DKK1*dickkopf-related protein 1, *MIP* macrophage inflammatory protein, *IL* interleukin, *sFRPs* secreted frizzled-related proteins, *TGF* transforming growth factor, *Gfi-1* growth factor independent 1, *HGF* hepatocyte growth factor, *RANKL* receptor activator of nuclear factor-κB ligand, *SDF* stromal-derived factor, *TNF* tumour necrosis factor, *BDNF* brain-derived neurotrophic factor, *lncRNA MEG3* long noncoding RNA maternally expressed gene 3, *DcR3* decoy receptor 3, *MMP-13* matrix metalloproteinase-13

In addition to the well-established role of osteoclast activation, it is now accepted that a markedly suppressed osteoblast activity contributes to the development of myeloma bone disease as well. Histomorphometric analysis of bone biopsies from patients with overt myeloma showed a reduced number of osteoblasts on bone surfaces adjacent to myeloma cells. As the progenitor cells of osteoblasts, MSCs from MM patients (but not MGUS patients) exhibited a significantly decreased osteogenic differentiation potential compared to MSCs from normal donors [69]. The suppression of osteogenic differentiation of MSCs in MM results from soluble factors produced by MM cells, which influence osteogenesis-related transcription factors and signalling pathways in MM-hMSCs (Table 2).

The communication between MM cells and MSCs also involves cell-to-cell interactions through VLA-4 on MM cells and VCAM-1 on MSCs, as demonstrated by the capacity of a neutralizing anti–VLA-4 antibody to reduce the inhibitory effects of MM cells on Runx2 activity [70]. The involvement of the VLA-4/VCAM-1 interaction in the development of bone lesions of MM has recently also been demonstrated using in vivo mouse models [71]. In addition to VLA-4/VCAM-1, other adhesion molecules appear to be involved in the inhibition of osteoblastogenesis by human MM cells, such as the neural cell adhesion molecule [72].

Wnt signalling has been found to play a critical role in the regulation of MSC osteoblastogenesis. There are two classes of extracellular antagonists of the Wnt signalling pathway, with distinct inhibitory mechanisms, acting either by binding directly to Wnt, such as secreted frizzled-related protein (sFRPs) and Wnt inhibitory factor 1 (WIF-1), or by binding to a part of the Wnt receptor complex, including certain members of the Dickkopf family. The involvement of Wnt signalling inhibitors in the suppression of osteoblast formation and function in MM has been investigated. Increasing Wnt signalling in the bone microenvironment in multiple myeloma with anti-DKK1 antibody or inhibition of glycogen synthase kinase-3β (GSK-3β) with lithium chloride resulted in the inhibition of myeloma bone disease as shown in a murine model of myeloma [73, 74]. MM cells can overexpress the Wnt inhibitor DKK1 compared to plasma cells from MGUS patients and to normal plasma cells [75]. High DKK1 levels in BM and peripheral blood sera of MM patients correlated with the presence of bone lesions [76]. MM cells also produce other Wnt inhibitors, including sFRP-2 and sFRP-3, which can inhibit osteoblast differentiation as well. Higher levels of these inhibitors are found in BM plasma of MM patients with bone lesions [76, 77]. Furthermore, Sclerostin, another Wnt pathway inhibitor with a mechanism of action similar to the related protein DKK1, was also found to be overexpressed in MM cells and involved in the osteoblast suppression [78, 79]. A recent study conducted by Bolzoni et al. [80] showed that MM cells could also inhibit osteogenic differentiation through the suppression of non-canonical Wnt co-receptor tyrosine kinase-like orphan receptor 2 (Ror2) expression in MSCs. Overexpression of Wnt family member 5 (Wnt5) or Ror2 by lentiviral vectors increased the osteogenic differentiation of hMSCs and blunted the inhibitory effect of MM [80].

In addition to Wnt signalling inhibitors, a few other soluble factors involved in the MM cell-mediated inhibition of osteoblast differentiation of MSCs have been identified. IL-7 can decrease runt-related transcription factor 2 (Runx2) promoter activity and the expression of osteoblast markers in osteoblastic cells. Higher IL-7 plasma levels were found in MM patients compared to normal subjects, and blocking IL-7 partially blunted the inhibitory effects of MM cells on osteoblast differentiation [70, 81]. Transforming growth factor-β (TGF-β) is a potent inhibitor of terminal osteoblast maturation and mineralization. Inhibition of TGF-β signalling can not only suppress myeloma cell growth but can also enhance osteoblast differentiation and inhibit bone destruction [82]. HGF is produced by MM cells, and its high levels in the BM plasma of MM patients correlates with those of alkaline phosphatase. HGF inhibits in vitro bone morphogenetic protein 2 (BMP2)-induced expression of alkaline phosphatase in both human and murine mesenchymal cells [83]. IL-3 has also been reported as a potential osteoblast inhibitor in MM patients. In both murine and human systems, IL-3 indirectly inhibited osteoblast formation in a dose-dependent manner, and IL-3 levels in the BM plasma from MM patients were increased in approximately 70% of patients compared to normal controls or MGUS patients [84]. In addition, it has been shown that myeloma cells promoted the release of activin A, a member of the TGF-β superfamily, by BM stromal cells. Activin A was found to inhibit osteoblast differentiation and bone formation. The BM plasma levels of activin A were also increased in MM patients with bone lesions, compared to those without bone lesions [85]. Treatment of 5T2MM-bearing mice with ActRIIA.muFc, a soluble form of the activin receptor, prevented myeloma-induced suppression of bone formation, loss of cancellous bone and the development of bone lesions [86]. Moreover, TNF-α is produced by MM cells and markedly increases IL-6 production by BM stromal cells, thereby preventing MM cell apoptosis and increasing MM cell proliferation [87]. TNF-α can also inhibit the proliferation of MSCs and induce apoptosis of mature osteoblasts [88]. Evidence was found that TNF-α produced by MM cells induces a higher growth factor independent 1 (Gfi-1) expression in MSCs resulting in repression of the Runx2 gene and osteoblast differentiation [89]. In addition, a new mechanism for MM cell-induced suppression of osteogenic differentiation has been proposed by Fu et al. [90] These authors demonstrated that MM cells can inhibit osteogenic differentiation of MSCs from healthy donors by rendering the osteoblasts sensitive to TNF-related apoptosis-inducing ligand (TRAIL)-induced apoptosis [90]. In addition, Vallet et al. [91] reported that MM cell-derived CCL3 exerted a strong inhibition of osteoblast function and that treating SCID-hu mice with an inhibitor of the corresponding chemokine (C-C motif) receptor 1 (CCR1) induced an upregulation of osteocalcin expression along with osteoclast downregulation.

Notch signalling has been reported to maintain BM mesenchymal progenitors in a more undifferentiated state by suppressing osteoblast differentiation. Our group found that the Notch pathway downstream genes hairy and enhancer of split-1(hes1), hairy/enhancer-of-split related with YRPW motif protein 1 (hey1), hey2, and heyL were considerably decreased in ND-hMSCs during osteogenesis. However, the expression of Notch signalling in MM-MSCs did not decrease to the level of ND-MSCs, suggesting that the Notch pathway remained over-activated in MM-MSCs. The addition of the Notch pathway inhibitor DAPT or Notch1 short interfering RNA (siRNA) could significantly enhance the impaired osteogenic differentiation ability of MM-MSCs in vitro [69].

As mentioned above, MM-hMSCs have a different miRNA expression profile compared to ND-hMSCs. We observed that miR-135b negatively regulates MSC osteogenesis. Noticeably, miR-135b was significantly upregulated in MM-hMSCs with low alkaline phosphatase (ALP) activity compared to ND-hMSCs. A miR135b inhibitor could enhance MM-hMSC osteogenic differentiation [13]. In addition, Zhuang et al. [92] demonstrated that MM-MSCs expressed lower levels of long noncoding RNA (lncRNA) maternally expressed gene 3 (MEG3) compared to those from normal donors during osteogenic differentiation. Gain- and loss-of-function studies demonstrated that lncRNA MEG3 played an essential role in the osteogenic differentiation of BM MSCs, partly by activating BMP4 transcription [92].

## MSC-based cell therapy in MM

MSCs possess different properties that might make them an attractive choice as cellular vehicles for cell-mediated gene therapy in human malignancies. MSCs can be easily genetically manipulated in vitro to carry anti-tumour agents to tumour sites based on their tumour-tropism migration ability. Several pre-clinical studies have applied this cell-mediated gene therapy strategy successfully in MM. Rabin et al. [47] observed in the KMS-12-BM MM mouse model that the presence of MM cells in the BM could attract infused MSCs, suggesting that BM-derived MSCs can be good candidates to deliver therapeutic transgenes to the MM environment in vivo. Hence, they engineered MSCs lentivirally with OPG in vitro and employed MSCs as a vehicle to deliver OPG in vivo to treat MM-induced bone lesions. The results showed that the systemic administration of OPG-expressing MSCs reduced osteoclast activation and trabecular bone loss in the vertebrae and tibiae of MM diseased animals. Sartoris et al. [46] also reported that the subcutaneous administration of interferon-α engineered MSCs significantly hindered the tumour growth and prolonged the overall survival in a mouse plasmacytoma model. Moreover, Ciavarella et al. [45] demonstrated in vitro that TRAIL-expressing adipose-derived MSCs could not only directly induce myeloma cell death but also synergistically potentiate the anti-myeloma activity of Bortezomib.

The capacity to target endogenous MSCs towards committed differentiation in vivo using pharmacological agents has recently been emphasized. Bortezomib is a clinically available proteasome inhibitor used for the treatment of multiple myeloma. It was incidentally observed that multiple myeloma patients treated with the drug have increased serum levels of bone-specific alkaline phosphatase. Giuliani et al. [93] reported in vivo and in vitro observations that both direct and indirect effects on the bone formation process could occur during bortezomib treatment, while Mukherjee et al. [94] further showed that bortezomib can induce MSCs to preferentially undergo osteoblastic differentiation, in part by modulation of the bone-specifying transcription factor Runx2 in mice. Mice implanted with MSCs showed increased ectopic ossicle and bone formation when recipients received low doses of bortezomib. This treatment increased bone formation and rescued bone loss in a mouse model of osteoporosis. Furthermore, osteoblasts and MSCs express the vitamin D receptor (VDR), which positively regulated osteogenic differentiation. Kaiser et al. [95] demonstrated that stimulation of VDR is another mechanism for the bortezomib-induced stimulation of osteoblastic differentiation, which suggests that supplementation with vitamin D of MM patients treated with bortezomib is crucial for optimal bone formation.

Histone deacetylase inhibitors (HDACis) are also considered to be promising drugs for the treatment of MM and other cancers. There is growing evidence that some HDACis, such as trichostatin A, valproic acid and sodium butyrate, could stimulate the osteogenic differentiation of MSCs and exhibit anabolic effects on the skeleton in addition to their anti-tumour effect [96–99]. Vorinostat (SAHA or ZolinzaTM) is a pan-inhibitor of class I and II HDAC proteins. However, two recent publications showed that high treatment frequency of Vorinostat (100 mg/kg, daily) caused bone loss in mice [100, 101]. It is unclear why Vorinostat apparently induces an opposite effect compared to other HDACis, but it was noticed in the study of McGee-Lawrence et al. [100] that a high treatment frequency also caused a significant toxicity (body weight loss) and even death. According to Campbell et al. [102], Vorinostat at 100 mg/kg daily intraperitoneally for 2 consecutive days per week already showed a marked decrease in MM tumour burden, and no further improvement of the anti-MM effect occurred when the frequency of drug treatment was increased to 5 consecutive days per week [102]. In our study, we observed that Vorinostat significantly increased in vitro the activity of ALP, the mRNA expression of osteogenic markers and matrix mineralization in BM-derived hMSCs from both normal donors and MM patients. Importantly, with a less frequent treatment regimen, we did not observe any decrease in bone formation in vivo in contrast to what previous publications showed [103]. These data suggest that with an optimized treatment regimen, Vorinostat can retain its anti-tumour effect without impairment of bone formation or even with a supportive effect on osteogenesis. Further in vivo work is needed to determine an optimal treatment strategy that can kill MM cells without impairing MSC function.

Bisphosphonates are widely used in the treatment of bone loss in cancers, but the observations about their effects on MSC differentiation towards osteoblast cells are controversial. Heino et al. [104] reported that zoledronic acid (ZA) could increase proliferation of rat mesenchymal stromal cells in vivo, but did not observe substantial effect on osteoblastic differentiation of MSCs. However, Hu et al. [105] found that ZA-pretreated murine BM-MSCs showed increased osteogenesis in vivo. The opposite results might be related to the concentration of ZA that was used for treatment. In the study of Hu et al. [105], it was demonstrated that non-toxic levels of ZA (0.5 μM) can upregulate the expression of the osteogenesis-related genes Alp, osterix and bone sialoprotein in MSCs, while at higher concentrations (5 and 10 μM) ZA inhibits the proliferation and osteogenic differentiation of BM-MSCs. This opposite effect has also been observed with other anti-MM drugs, such as the HDCA inhibitor Vorinostat [103] which suggests that the drug concentration as well as the frequency and timing of treatment might significantly influence MSC differentiation in the BM environment.

## Conclusions and perspectives

MSCs only constitute a small population of adult stem cells mainly found in the BM but play an important role in a number of malignant diseases, particularly in MM and MM-induced bone disease. Compared with their normal counterparts, MSCs are abnormal in MM patients at both the genomic and cytokine secretion levels. Moreover, they show an impaired osteogenic differentiation ability. At which level these abnormalities are intrinsic or acquired through contact with the myeloma clone remains to be determined. Several in vitro studies have shown that some anomalies can be induced by exposing normal MSCs to myeloma cells. On the other hand, several defects remain detectable in MM bone marrow–derived MSCs, even when the cells are cultured for a prolonged period of time without myeloma cells. This indicates that if anomalies are myeloma cell induced, they are at least not immediately reversible in the absence of tumour cells.

Multiple factors are involved in MM cell-mediated inhibition of MSC osteogenic differentiation. Although blockade of soluble factors such as IL-3, IL-7, sFRP-2 and HGF, as well as anti-DKK1 and anti-CCR1 treatments, can partially improve bone disease in MM, no treatment can totally correct the impairment of MM-induced MSC osteogenesis, indicating that more complex mechanisms may be involved. Drug targeting of MSCs has been proposed as a strategy for repairing MM-induced bone lesion. Emerging data indicate that the proteasome inhibitors such as bortezomib may regulate MSC osteogenic differentiation, stimulate bone formation and control MM bone disease. In addition to bortezomib, other new MM drugs should be further investigated for their potential MSC osteogenesis stimulatory effect.

MSCs possess numerous properties that might make them an attractive choice as a vehicle for gene therapy in human malignancies. MSCs can be easily genetically manipulated in vitro and carry anti-tumour agents to tumour sites based on their tumour-tropism migration ability. Several studies have applied this cell-mediated gene therapy strategy successfully against different tumour types. Recently, Liu et al. [106] also used mechanosensitive promoter-driven MSC-based vectors to deliver cytosine deaminase which could selectively target cancer metastases. With regard to MM, the application of gene-modified MSCs for the treatment of MM is still in its infancy. Only two reports have described OPG-expressing MSCs for the treatment of MM bone lesion and TRAIL-expressing MSCs to kill MM cells.

Although the use of MSCs in cell-based therapies shows great promise in many tumour types, and MSC infusion is a promising approach to support haematopoietic recovery and to control graft versus host disease (GVHD) in patients after allogeneic haematopoietic stem cell transplantation, the application of MSCs in MM should be handled with extreme caution. Our data suggest that MSCs, as the progenitors of most BM cells, could favour MM growth in vitro and in vivo and that MSC-based cytotherapy might introduce a potential risk for MM disease progression or relapse. Ning H et al. [107] recently reported the outcome of their pilot clinical study indicating that cotransplantation of MSCs in haematologic malignancy patients can prevent GVHD. However, the relapse rate was obviously higher than the control group, which may be explained by MSC immunomodulatory properties [107]. Although the studies from our team and other groups showed that BM-derived MSCs have the potential to contribute to myeloma disease, MSCs from other sources, such as umbilical cord and adipose tissue, were found to inhibit MM growth or to have no significant effect [43, 45]. Compared to BM-MSCs, MSCs derived from UC and adipose were shown to exhibit distinct biological properties related to expansion capacity, gene expression, osteogenesis capacity and cytokine/chemokine secretion potentials [43, 108]. Therefore, the therapeutical use of MSC from non-BM tissue sources in MM is worthy of being further investigated.

In addition, there are some other issues that have to be addressed in future research and therapeutical applications of MSCs. Several groups described that murine MSCs can undergo spontaneous transformation in vitro which is associated with increased telomerase activity, p53 loss, oncogene activation, or/and accumulated chromosomal instability [109–111]. This risk of in vitro transformation or other culture-induced abnormalities must be considered when these cells are used as a model to study in vivo and/or in vitro the biological properties of “normal” MSCs. In contrast, studies using human MSCs did not provide any evidence so far that culture expansion can result in malignant transformation and therefore the safety of MSC therapy is currently considered to be acceptable. On the other hand, prolonged in vitro expansion might alter biological characteristics of MSCs. Therefore, it is important to identify optimal culture conditions which allow cell expansion without affecting basic features like the primary “stemness” and differentiation potential as well as the homing properties of MSCs. Traditionally, foetal bovine serum has been utilized as the main source of growth supplement for MSC culture in clinical protocols. However, the use of an animal-derived product might have potential safety concerns for the recipients of MSC therapy, including possible infections and severe immune reactions. Some alternative animal-free culture conditions have been developed, including human platelet lysates and chemically predefined serum-free culture media, which could retain all necessary characteristics attributed to MSC for potential therapeutic use [112–114]. However, the long-term in vivo safety and efficacy require further investigation.

Collectively, MSCs are not just passive by-standers in the MM BM microenvironment. MSCs can be considered both as a possible therapeutic tool or a target for MM patients. The crosstalk between MSCs and MM cells is very important for MM cell growth and MM-induced bone disease. Using MSCs as cell carriers to deliver anti-tumour factors or targeting MSCs themselves might lead to promising MM therapies, but their potential risk must be further examined.
